# Multiple In Vivo Biological Processes Are Mediated by Functionally Redundant Activities of *Drosophila mir-279* and *mir-996*

**DOI:** 10.1371/journal.pgen.1005245

**Published:** 2015-06-04

**Authors:** Kailiang Sun, David Jee, Luis F. de Navas, Hong Duan, Eric C. Lai

**Affiliations:** 1 Sloan-Kettering Institute, Department of Developmental Biology, New York, New York, United States of America; 2 Neuroscience Program, Weill Graduate School of Medical Sciences, Cornell University, New York, New York, United States of America; 3 Molecular Biology Program, Weill Graduate School of Medical Sciences, Cornell University, New York, New York, United States of America; The University of North Carolina at Chapel Hill, United States of America

## Abstract

While most miRNA knockouts exhibit only subtle defects, a handful of miRNAs are profoundly required for development or physiology. A particularly compelling locus is *Drosophila mir-279*, which was reported as essential to restrict the emergence of CO_2_-sensing neurons, to maintain circadian rhythm, and to regulate ovarian border cells. The *mir-996* locus is located near *mir-279* and bears a similar seed, but they otherwise have distinct, conserved, non-seed sequences, suggesting their evolutionary maintenance for separate functions. We generated single and double deletion mutants of the *mir-279* and *mir-996* hairpins, and cursory analysis suggested that miR-996 was dispensable. However, discrepancies in the strength of individual *mir-279* deletion alleles led us to uncover that all extant *mir-279* mutants are deficient for mature miR-996, even though they retain its genomic locus. We therefore engineered a panel of genomic rescue transgenes into the double deletion background, allowing a pure assessment of miR-279 and miR-996 requirements. Surprisingly, detailed analyses of viability, olfactory neuron specification, and circadian rhythm indicate that miR-279 is completely dispensable. Instead, an endogenous supply of either *mir-279* or *mir-996* suffices for normal development and behavior. Sensor tests of nine key miR-279/996 targets showed their similar regulatory capacities, although transgenic gain-of-function experiments indicate partially distinct activities of these miRNAs that may underlie that co-maintenance in genomes. Altogether, we elucidate the unexpected genetics of this critical miRNA operon, and provide a foundation for their further study. More importantly, these studies demonstrate that multiple, vital, loss-of-function phenotypes can be rescued by endogenous expression of divergent seed family members, highlighting the importance of this miRNA region for in vivo function.

## Author Summary

Amongst the small number of miRNA knockouts that exhibit substantially overt phenotypes, mutants of *Drosophila mir-279* are notable. Previous studies have uncovered its essential requirements in a range of developmental and behavioral assays. Surprisingly, we find that the phenotypes attributed to *mir-279* deletions depend on the unanticipated loss of expression of the downstream locus *mir-996*, whose genomic locus is retained in extant *mir-279* mutants. These miRNAs share their seed regions but are divergent elsewhere in the mature sequences. We use precise genetic engineering to show that a single endogenous copy of either *mir-279* or *mir-996* can fully rescue viability, olfactory neuron, and circadian rhythm defects of double deletion animals. These data and genetic reagents set a new foundation for developmental and behavioral studies of this critical miRNA locus. More generally, these data demonstrate that multiple loss-of-function phenotypes can be rescued by endogenous expression of divergent seed family members, highlighting the importance and potentially sufficiency of this region for in vivo function.

## Introduction

microRNAs (miRNAs) are ~22 nucleotide (nt) regulatory RNAs derived from hairpin precursors [1], and there are 100s ~ 1000 miRNA loci in well-studied animal genomes [2]. As animal miRNAs regulate targets exhibiting as little as 7 nt of complementarity to their 5' regions (principally nts 2–8, known as the "seed region"), they coordinate large regulatory networks [3]. Collectively, the developmental and physiological impacts of miRNA-mediated regulation are extensive and substantial [4,5].

The first miRNAs discovered, nematode lin-4 and let-7, exhibit strong developmental defects and have key individual targets that mediate substantial aspects of their phenotype [6–8]. As well, gain-of-function neural phenotypes associated with loss of 3' UTR elements from Notch target genes identified the functional logic of miRNA binding sites and highlighted additional key targets of miRNAs [9–11]. On the other hand, it is now well-appreciated that knockouts of individual miRNA genes frequently lack substantial phenotypes [12,13], and that the typical range of miRNA-mediated repression is modest [14,15]. Such findings have been interpreted to reflect that miRNAs are usually for "fine-tuning" or "robustness" of gene expression [5,16], but perhaps dispensable for major aspects of development, metabolism and behavior.

Nevertheless, a handful of animal miRNA mutants exhibit dramatic phenotypes in one or more settings. A particularly compelling example is miR-279 [17,18]. This miRNA is one of just a few loci across all animals, including nematode *lin-4* [19], *let-7* [8], *lsy-6* [20], and mouse *mir-96* [21,22], to have emerged from forward loss-of-function genetics, attesting to the strength of its mutant phenotype. By comparison, virtually every other miRNA studied in intact animals originated from gain-of-function screening or a directed knockout (although clearly in some cases these proved to have substantial effects).

A mutant of *mir-279* initially emerged from a genetic screen for altered patterning of olfactory neurons, yielding a line with ectopic CO_2_-sensing neurons in the maxillary palp [18]. This was associated with a transposon insertion near *mir-279*, which was phenocopied by multiple *mir-279* deletion alleles. In this setting, the transcription factors encoded by *nerfin-1* and *escargot* are critical miR-279 targets [18,23]. Subsequent studies defined additional functions of miR-279, including to mediate normal circadian activity [24] and for specification and migration of border cells [25]. Curiously, these other settings were associated with deregulation of JAK-STAT signaling, although via different mechanisms. miR-279 restricts the JAK-STAT ligand unpaired in circadian pacemaker cells [24], whereas in ovarian border cells it represses the transcription factor STAT [25]. Altogether, these studies highlight diverse requirements for miR-279 in development and behavior.

The *mir-996* locus was later identified in the vicinity of *mir-279* and shown to encode a similar seed, but they otherwise have distinct mature sequences and were originally suggested to derive from separate genes [26,27]. Notably, miR-996 has not been implicated in any biological processes, since available *mir-279* deletion alleles do not affect the *mir-996* locus, and *mir-279* mutant phenotypes can be rescued by a genomic transgene that contains only *mir-279* and lacks *mir-996* sequence [18]. Indeed, the deep conservation of divergent non-seed regions of miR-279 and miR-996, and the observation that *mir-279* is ancestral and that *mir-996* emerged more recently during arthropod evolution [28], suggest that miR-996 may have neofunctionalized from miR-279 to acquire some distinct activity.

In this study, we generated single and double mutants of *mir-279* and *mir-996*, and cursory examination suggested that miR-996 was dispensable for overt development and behavior, while miR-279 was essential. However, our studies unexpectedly reveal that all *mir-279* single deletion mutants affect the expression of miR-996. In essence, then, all studies of *mir-279* mutants to date [18,23–25] have effectively been of double mutants. To remedy this, we engineered a defined set of backgrounds, using recombineered knockin/knockout transgenes introduced into the double deletion, to distinguish individual miRNA sequence requirements from dosage effects. We find that miR-996 contributes essential function in all known biological settings of miR-279 activity, such that a single genomic dose of either *mir-996* or *mir-279* provides nearly wildtype rescue to double deletion mutants in all characterized neural and non-neural settings. These data strongly support the notion that the seed region is the major determinant of *in vivo* miRNA function in animals.

## Results

### Generation of single and double deletion alleles of the *mir-279* and *mir-996* loci

**Fig 1:**
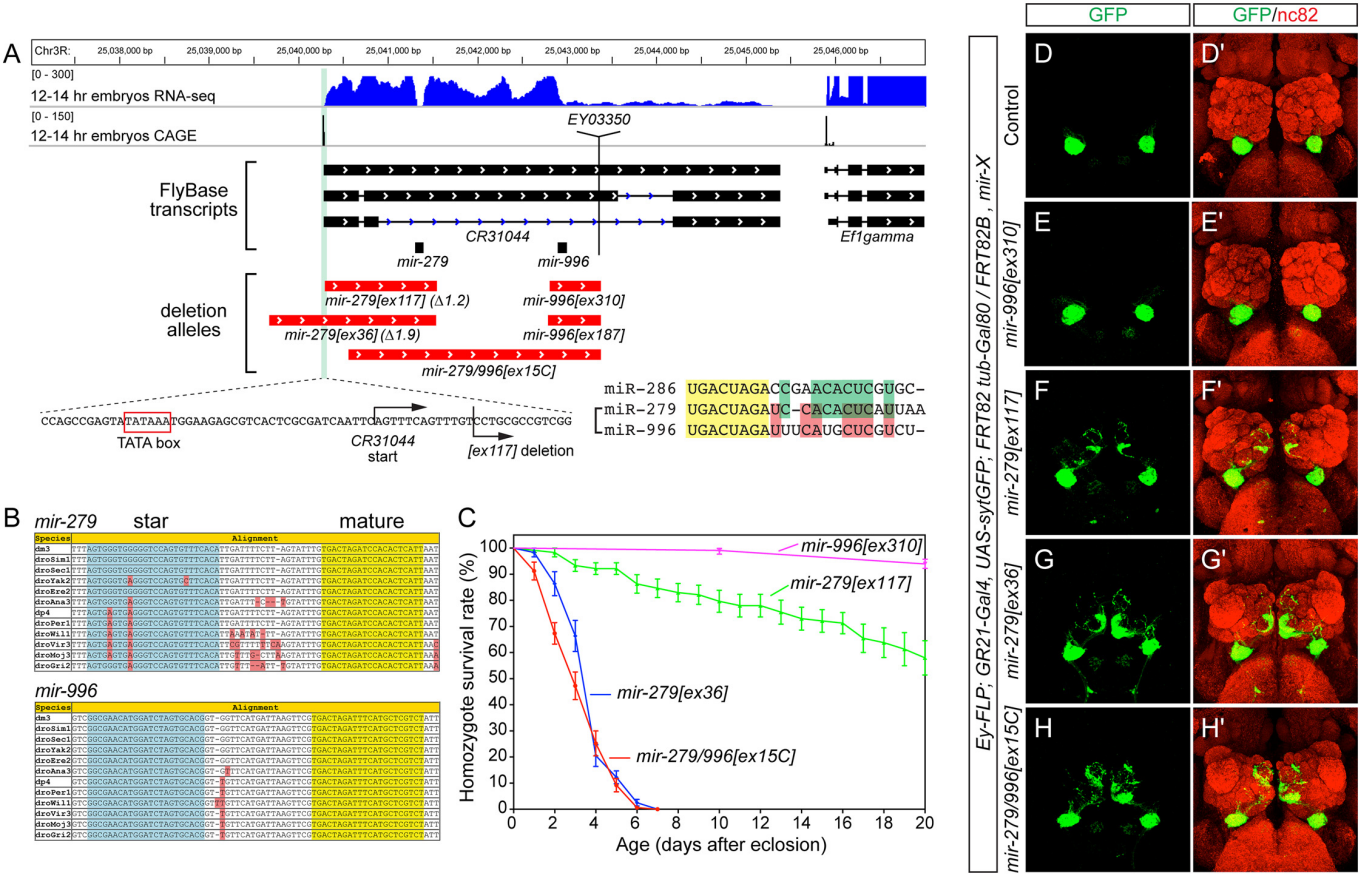
The *mir-279/996* locus and phenotypes of single and double deletion alleles. (A) Annotation of the *mir-279/996* region in the *Drosophila* R5/dm3 version. RNA-Seq data (in blue) reveals characteristic "dropoffs" in read density at the Drosha cleavage sites for *mir-279* and *mir-996*. CAGE data (in black) reveals a single peak in the *mir-279/996* region located downstream of a TATA box, likely representing the start of a shared primary *mir-279/996* transcript (i.e., *CR31044*). The genomic regions deleted in five *mir-279/996* alleles are shown in red. Zoom-in of the 5' breakpoint of the *mir-279[ex117]* deletion shows it removes sequence just downstream of the *CR31044* TSS, leaving the putative TATA box intact. The mature sequences of three members of the miR-279 seed family are shown; note that miR-286 is encoded elsewhere in the genome but is more related to miR-279 than is miR-996. (B) Alignments of *pri-mir-279* and *pri-mir-996* demonstrate their distinct mature miRNAs are perfectly conserved across across 12 Drosophilids. (C) Distinct lifespan of various *mir-279/996* deletion alleles. n = 100 for *mir-996[ex310]* mutants, n>150 were assayed for the different *mir-279* mutant alleles; equal numbers of males and females were included. Error bars represent SEM. (D-H) MARCM analysis of GR21+ neurons CO_2_-sensing neurons. The tester stock genotype is shown, where "X" refers to the mutations as labeled. (D, D') The normal projection pattern of GR21+ neurons to ventral glomeruli in control clones. (E, E') Deletion of *mir-996* does not perturb CO_2_-sensing neurons. (F, F') *mir-279[ex117]* clones exhibit substantial ectopic projections to medial glomeruli, whereas *mir-279[ex36]* clones (G, G') and *mir-279/996[ex15C]* clones (H, H') exhibit more extensive misprojections.

The *mir-996* hairpin is located ~1.5 kb downstream of the *mir-279* hairpin (Fig 1A). While they share seed regions, the rest of their mature regions are distinct and well-conserved (Fig 1B). When we initially annotated *mir-996*, its hairpin was embedded in the annotated gene *CG31044* (FlyBase Release 5.12), which was predicted to encode a short, non-conserved, protein. At the time, we hypothesized that *CG31044* might represent the primary transcript for *mir-996*, distinct from *mir-279* [26]. A similar inference of separate transcription units for these miRNAs was reported in a concurrent study [27]. Phylogenomic tracing indicates that *mir-279* is ancestral and that *mir-996* has adopted a derived sequence [28]. A third seed member, *mir-286*, is genomically unlinked from the *mir-279/996* cluster and moreover deployed in a spatially and temporally distinct manner, being essentially restricted to early embryogenesis [26,29,30] (S1 Fig). Nevertheless, mature miR-279 is more similar to miR-286 than it is to miR-996 (Fig 1A). This suggests that miR-279 and miR-996 are selected for distinct sequences, presumably related to some separable functions.

Our previous efforts yielded two alleles in this region, *[ex117]* and *[ex36]*, that delete *mir-279* but spare the *mir-996* locus (Fig 1A). As the phenotypes of these mutants were rescued by a ~3kb genomic transgene bearing only *mir-279*, and lacked *mir-996* sequence [18], *mir-279* appeared to be causal. Nevertheless, we were interested to assess whether miR-996 contributes to any biological settings known to depend on miR-279. We screened excisions of a P element inserted downstream of *mir-996*, and recovered two small deletions (*[ex187]* and *[ex310]*) that selectively remove this locus. We also recovered a longer deletion (*[ex15C]*) that removes both *mir-279* and *mir-996* loci, thus establishing an apparent allelic series of single and double mutants of these miRNAs (Fig 1A).

Both *mir-996* single deletions were homozygous viable and lacked obvious morphological or behavioral defects. We measured the lifespan of *mir-996[ex310]* mutants and this was normal (Fig 1C). We also analyzed the projections of GR21+ olfactory neurons. In wildtype, these CO_2_-sensing neurons are present only in the antenna and they project to ventral glomeruli. We visualized these in control *ey-FLP; FRT82B* MARCM clones generated in the *GR21-Gal4*, *UAS-synaptotagmin-GFP* background, and stained for GFP-labeled projections to brains that were counterstained with nc82 (Fig 1D). The GR21+ projections of *mir-996* deletion MARCM clones were identical to wildtype (Fig 1E). In contrast, *mir-279* deletions induced ectopic medial projections (Fig 1F and 1G), as described [18], and reflected the generation of ectopic CO_2_-sensing neurons in the maxillary palp.

Our newly generated *mir-279*/*996* double deletion mutant *[ex15C]* exhibited similar gross phenotypes as *mir-279[ex36]*, with respect to lifespan (Fig 1C) and ectopic GR21+ projections (Fig 1H). As well, the pharate lethality of homozygous *[ex15C]* mutants was well-rescued by the *mir-279*-only genomic transgene. Altogether, these findings were consistent with an interpretation that miR-279 is primarily responsible for essential genetic requirements of this two-miRNA locus.

### Unanticipated effects of *mir-279* deletions on *mir-996* expression

While the initial genetic data were reasonably explained by phenotypic dominance of miR-279, certain other observations remained difficult to account for. Perhaps most germane was the fact that the *mir-279[ex117]* and *mir-279[ex36]* single deletions, both null for *mir-279*, exhibited distinct viability. While *mir-279[ex36]* is lethal within a few days of eclosion, *mir-279[ex117]* adults can eclose and survive for weeks with optimal care, despite their locomotor difficulties (Fig 1C). The discrepancy of these alleles was exploited in the circadian rhythm studies of Sehgal and colleagues; such behavioral studies require adult viability of at least one week [24]. Their analyses utilized a stock of *mir-279[ex117]* that had been outcrossed to remove potential second-site aberrations.

A plausible model was that *mir-279[ex36*] bears an unlinked mutation responsible for its stronger defects. However, we were unable to recover homozygous *mir-279[ex36]* stocks that survived longer, even after extensive outcrossing of this mutant chromosome. In addition, flies carrying *mir-279[ex36]* in trans to a deficiency of the region showed the same gross phenotypes. Finally, we could rescue the viability and locomotor behavior of this mutant using the 3kb *mir-279*-only genomic transgene. Taken together, these findings suggested that phenotypic differences between *mir-279[ex117]* and *mir-279[ex36]* must reside extremely close to the *mir-279* hairpin. For example, there might theoretically be another non-coding function of the primary *mir-279* transcript, or even perhaps a peptide encoded by this region.

**Fig 2:**
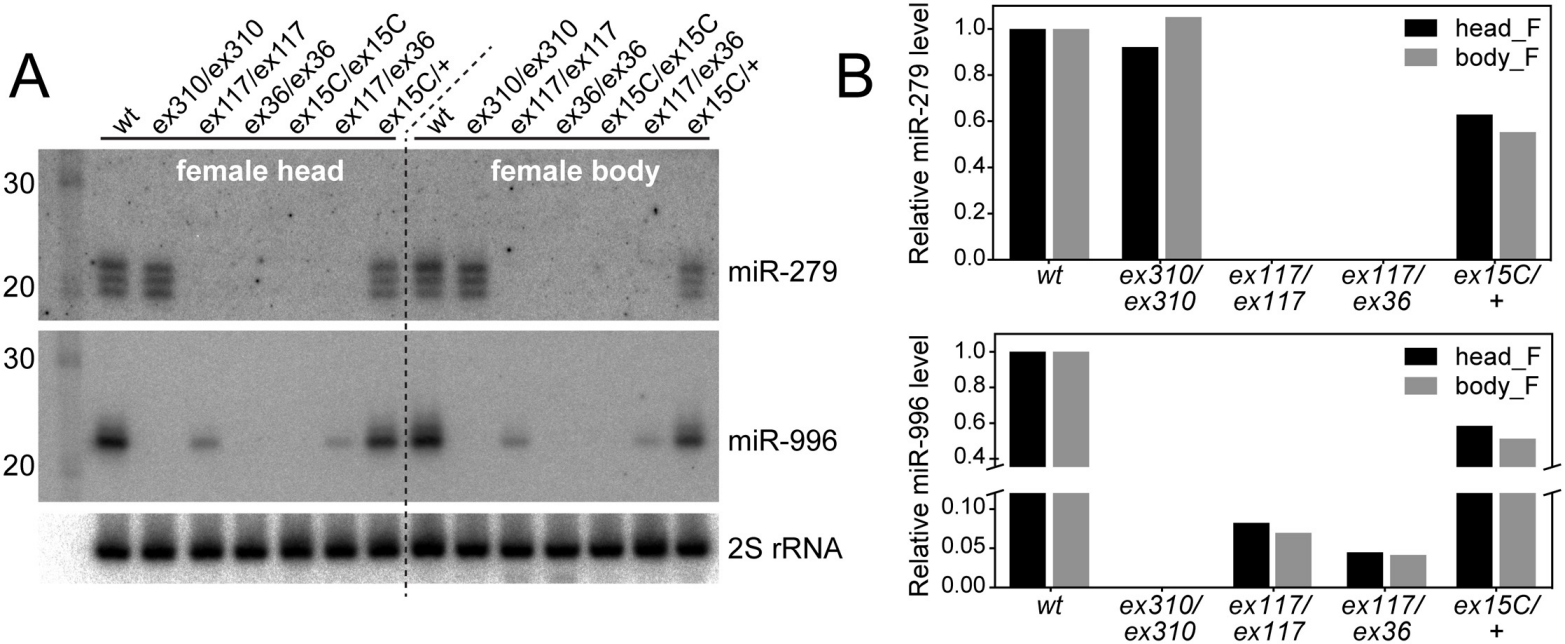
Severe loss of mature miR-996 expression in *mir-279* deletion alleles. (A) Northern blots of miR-279 and miR-996 in various *mir-279* and *mir-996* homozygous or trans-heterozygous allele combinations. In *mir-279* alleles *[ex117]* and *[ex36]* that retain the *mir-996* genomic DNA, the levels of mature miR-996 are strongly diminished (*[ex117]*) or nearly undetectable (*[ex36]*). *mir-996[ex310]* is a deletion of the *mir-996* region that does not affect *mir-279* and *mir-279/996[ex15C]* deletes both miRNAs. (B) Quantifications of mature miR-279 and miR-996 levels. Homozygous *[ex117]* mutants expressed ~10% of the wild type level of miR-996 and *[ex117/ex36]* transheterozygous mutants expressed ~5% of miR-996. Note that the expression level for both miRNAs is copy-number dependent, since heterozygous *mir-279/996[ex15C]* flies expressed roughly half of both miRNAs compared to wild type animals.

However, another scenario we considered was that miR-996 might be affected by available miR-279 alleles. Northern analysis of small RNAs in different *mir-279* and *mir-996* alleles confirmed this hypothesis. The *mir-996[ex310]* homozygous mutant lacked mature miR-996, validating its nature as a null allele and demonstrating specificity of the miR-996 probe; this mutant expressed miR-279 normally (Fig 2A). Both *mir-279* "single" alleles and the *mir-279/996* double deletion failed to express mature miR-279, as expected, but all of these mutants also proved deficient for miR-996. *mir-279[ex117]* expressed <10% the normal level of miR-996, and *mir-279[ex36]* did not detectably express miR-996 (Fig 2B). We obtained similar results by examining different female tissues (Fig 2) as well as male tissues (S2 Fig). Therefore, we conclude that the available *mir-279* "single" mutants are unexpectedly also strong or nearly null alleles of *mir-996*.

Consideration of current modENCODE transcriptomic data at the *mir-279/996* region proved informative (Fig 1). Our small RNA analyses showed that miR-279 and miR-996 belong to the same expression cluster across diverse *Drosophila* tissue and cell line small RNA libraries [31], indicating their coordinate deployment. Inspection of companion transcriptome data [32] revealed relatively continuous, although graded, levels of RNA-seq reads across the entire locus, consistent with the notion of a single primary *mir-279*/*996* transcript. It is commonly observed in *Drosophila* that 3' fragments of Drosha-cleaved primary transcripts are less stable than 5' Drosha fragments [33,34], and evidently at the *mir-279*/*996* locus, the 3'-most fragment of its primary transcript is least stable of them all (Fig 1A). On the basis of such transcriptome data, the provenance of *CR31044* was expanded in the most recent FlyBase release (5.47), such that it now includes both *mir-279* and *mir-996* (Fig 1A).

Analysis of capped analysis of gene expression (CAGE) data revealed a 5' transcription start site ~1kb upstream of the *mir-279* hairpin. This lies <30nt downstream of a typical TATA box sequence (GTATATAAA), suggesting that as the promoter for the *mir-279/996* transcription unit. The deletion extents of the "*mir-279*" alleles, relative to the transcription start, were notable. *mir-279[ex36]* removes sequence upstream of the promoter, presumably explaining why this allele strongly compromises expression of the downstream miRNA. On the other hand, *mir-279[ex117]* deletes to within 14 nt of the transcription start site (Fig 1A), which does not abolish, but apparently debilitates expression and/or processing of the intact *mir-996* hairpin. Altogether, these molecular observations are consistent with our genetic inference that both miRNAs may contribute to mutant phenotypes uncovered by chromosomal aberrations of the region.

### Generation of genetically defined miR-279 and miR-996 single and double mutants

**Fig 3:**
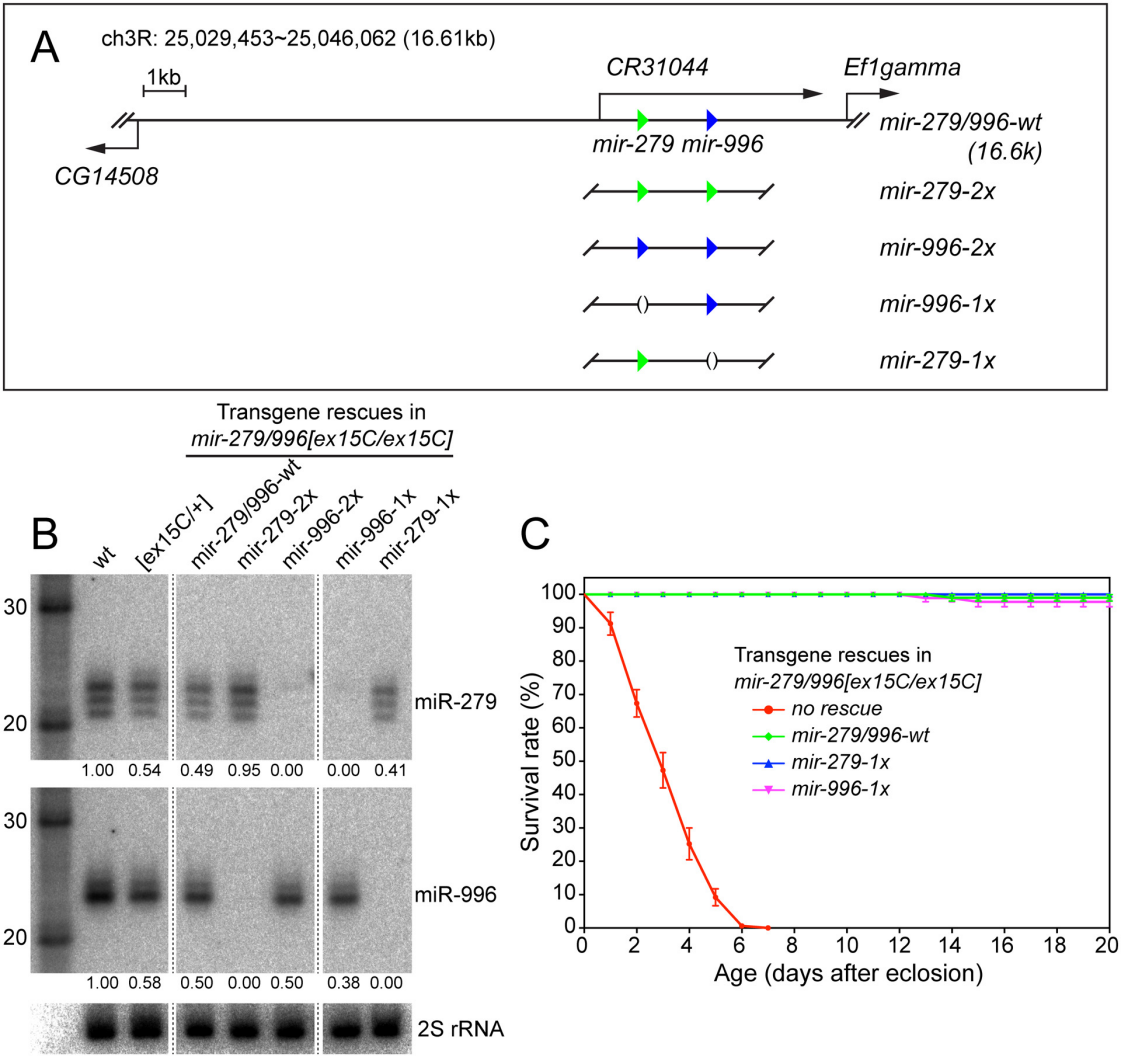
Modified genomic transgenes to assess individual miR-279/996 functions. (A) In the 16.6kb *mir-279*/*996* rescue transgene, the 5' end extends to cover a portion of the upstream *CG14508* gene and 3' end extends into the downstream *Ef1gamma* gene. Green and blue triangles represent *mir-279* and *mir-996* hairpins, respectively. The wildtype genomic fragment was modified to replace the *mir-279* and *mir-996* hairpins with either a deletion or the non-cognate miRNA. (B) Northern blots verify the expression of miR-279 and miR-996 from different genomic transgenes that were introduced into *mir-279/996[ex15C]* double mutant homozygous animals. RNA samples were extracted from whole flies carrying one copy of individual transgenes. Intensities of miR-279 and miR-996 expression were quantified by normalization to homozygous wild type and marked below each lane. *mir-996-2x* expressed only comparable amount of miR-996 as *mir-279/996-wt* and *mir-996-1x* transgenes. (C) Rescue of the lifespan defect in *mir-279/996[ex15C]* double mutants by 16.6kb transgenes. The *mir-279/996[ex15C]* data shown here are the same as plotted in Fig 1C. For each genotype, 100 flies including equal number of males and females were assayed; error bars represent SEM.

Since the available allelic series did not permit assessment of phenotypes caused by specific loss of miR-279, we sought an alternative strategy to analyze "clean" *mir-279* and *mir-996* mutant backgrounds. To do so, we recombineered a genomic transgene that includes the full 16.6kb intergenic region between the upstream (*CG14508*) and downstream (*Ef1gamma*) protein-coding genes (Fig 3A), and thus may be expected to confer full miRNA rescue. To permit direct comparison with variant forms, we used the phiC31 system to integrate transgenes into a common genomic site. The wildtype 16.6 kb transgene restored accumulation of mature miR-279 and miR-996 (Fig 3B) and fully rescued viability of *mir-279/996[ex15C]* double deletion homozygotes (Fig 3C). Using this genomic fragment, we then generated a series of mutant transgenes in which we specifically deleted 100bp covering either the *mir-279* or *mir-996* hairpins (*1x-mir-279* and *1x-mir-996*, which essentially serve as *mir-996-KO* and *mir-279-KO* transgenes, respectively) or replaced either miRNA with the non-cognate hairpin (*2x-mir-279* and *2x-mir-996*) (Fig 3A).

We placed one copy of each transgene into *mir-279/996[ex15C]* homozygotes, and performed Northern blotting for the two miRNAs from adult females. These tests demonstrated specific expression of mature miR-279 and miR-996 in the designated genotypes (Fig 1A). This confirmed their status as a bona fide panel of single mutants of the *mir-279/996* locus, an allelic series that was not functionally fulfilled by corresponding single genomic deletions. For reasons that are not apparent, the amount of mature miR-996 from the transgenic copies, especially the reprogrammed allele, was not as robust as the endogenous chromosomal locus (Fig 3B). Nevertheless, each of the four transgenes fully rescued adult viability of *mir-279/996* double deletion backgrounds. This provided an initial view into the complicated genetics of this locus. Strikingly, endogenous expression of only a single copy of either *1x-mir-279* or *1x-mir-996* transgenes in the double mutant, thus recapitulating full knockout of either miRNA on top of heterozygosity for the other, was sufficient to rescue adult viability (Fig 3C). This indicates that there is no essential requirement for the unique miR-279 sequence, and that one allele of either *mir-279* or *mir-996* supports normal viability of *Drosophila*.

We proceeded to subject these engineered miRNA backgrounds to detailed phenotypic study, to ascertain the extent to which defects previously attributed to miR-279 might actually depend on the joint function of miR-279 and miR-996.

### Both miR-279 and miR-996 mediate specification of CO_2_-sensing neurons

Under MARCM clonal conditions, *mir-996* single hairpin deletions exhibit normal specification of CO_2_-sensing neurons within the antenna, and these project to ventral glomeruli in the central brain (Fig 1D and 1E). In contrast, *mir-279* single hairpin deletions, which we now recognize as deficient for mature miR-996, exhibit ectopic CO_2_-sensing neurons in the palp, and these project to medial glomeruli. In particular, *mir-279[ex117]* (which retains some expression of miR-996) exhibits a weaker GR21 phenotype than *mir-279[ex36]* or *mir-279/996[ex15C]* (which are nearly null or definitively null for both miRNAs, respectively) under clonal conditions (Fig 1F–1H).

**Fig 4:**
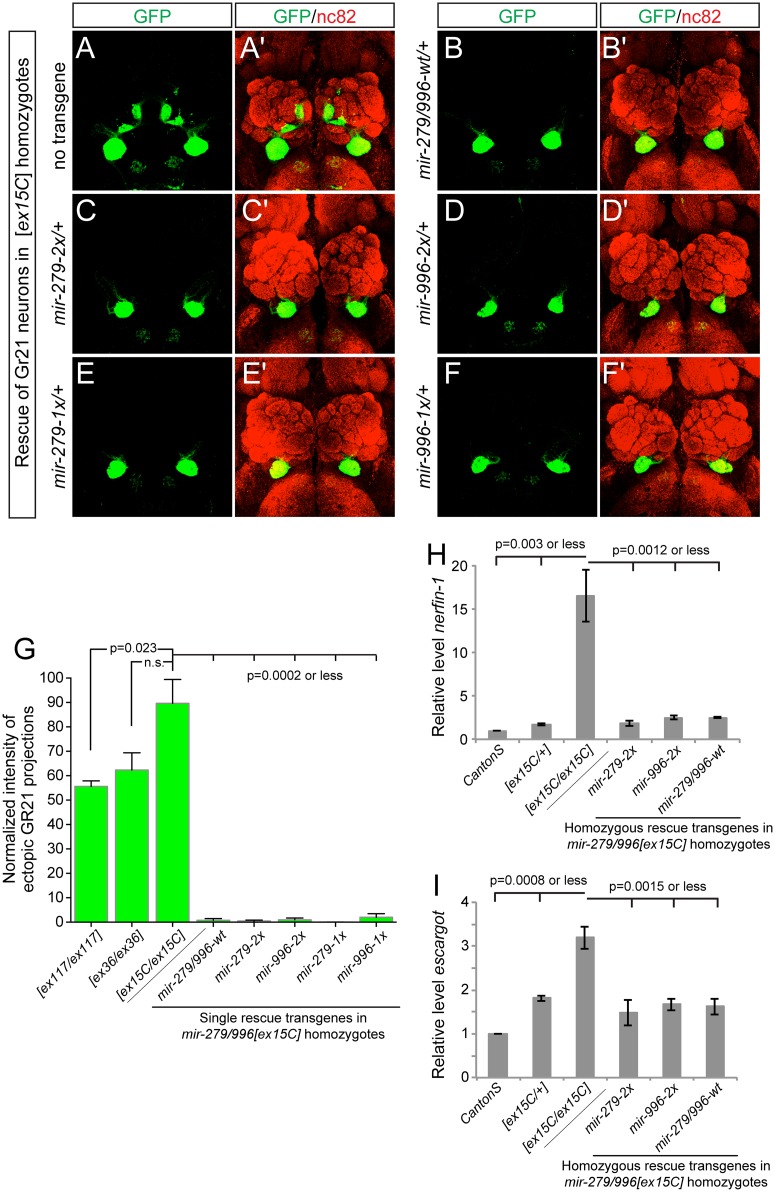
miR-996 restores proper CO_2_-sensing neurons to *mir-279/996* double mutants. (A-F) Representative images of GR21 projections in brains from *mir-279/996[ex15C]* whole animal mutants and rescued backgrounds bearing one copy of each 16.6 kb genomic transgene. Genotype for the mutant is: *Gr21-Gal4*, *UAS-syt-GFP/+; mir-279/996[ex15C]*, and rescued genotypes are: *Gr21-Gal4*, *UAS-syt-GFP/mir[rescue]; mir-279/996[ex15C]*. (A, A') Double deletion *mir-279/996[ex15C]* homozygote. (B-F) Rescue of *mir-279/996[ex15C]* homozygotes by a copy of the wildtype transgene (B, B'), by a single *mir-279*-only transgene (C, C'), by a single *mir-996*-only transgene (D, D'), by a two-copy *mir-279*-only transgene (E, E'), and by a two-copy *mir-996*-only transgene (F, F'). (G) Quantification of ectopic Gr21 axon projection normalized to the normal ventral projection. 4–6 brains were analyzed for each genotype, error bars represent SEM, and p-values calculated by Student's two-tailed T-test, equal variance. (H-I) qPCR assays show elevated *nerfin-1* (H) and *escargot* (I) transcript levels in *mir-279/996[ex15C]* homozygous heads. These defects are comparably rescued by *mir-279-2x* or *mir-996-2x* transgenes as they are by wildtype *mir-279/996* genomic transgene, to target levels that are slightly higher than in *Canton S* heads but similar to *[ex15C]/+* heads. Error bars represent SD, p-values calculated using unpaired T-tests from the combined data of independent, triplicate qPCR assays.

These observations strongly hinted that endogenous miR-996 contributes to suppression of CO_2_-sensing neurons in the maxillary palp. We sought to test this under non-clonal conditions, which are expected to be more sensitive than clonal conditions, which may potentially be rendered less potent by perdurance. It is more difficult to obtain *mir-279/996[ex15C]* homozygotes compared to MARCM mutant adults, but these proved to exhibit strong and fully penetrant GR21 projection phenotypes (Fig 4A). In fact, under these non-clonal conditions, this genuine double deletion mutant exhibited slightly stronger phenotypes than either of the shorter deletions that physically remove only *mir-279* but compromise *mir-996* expression (Fig 4G).

The *mir-279/996[ex15C/ex15C]* phenotype was fully rescued by a single insertion of the wildtype 16.6kb *mir-279/996* transgene, validating its status as a fully functional genomic fragment (Fig 4B). Moreover, single insertions of either "2x" transgene, in which *mir-279* was substituted for *mir-996*, and vice versa, also provided complete rescue of the ectopic CO_2_-sensing neurons (Fig 4C and 4D). In more stringent assays, we then showed that single insertions of either "1x" transgenes, recapitulating *mir-279*-knockout and *mir-996*-knockout conditions, similarly provided essentially full suppression of the double deletion phenotype (Fig 4E and 4F). We quantified these rescues, expressed as the relative amounts of ectopic GFP+ projections in the brain, in Fig 4G.

The ectopic CO_2_ neuron phenotype was shown to be driven by derepression of specific miR-279 targets, namely the transcription factors encoded by *nerfin-1* and *escargot* [18,23]. Although CO_2_-sensing neurons comprise only a small number of cells in the nervous system, we were able to detect massive derepression of *nerfin-1* (Fig 4H) and substantial upregulation of *escargot* (Fig 4I) transcripts in whole *mir-279/996[ex15C/ex15C]* adult heads, relative to *Canton S* control heads. Based on the degree of misregulation, we infer that miR-279/996 must regulate *nerfin-1* outside of the CO_2_-sensing apparatus. In mutant heads bearing either wildtype *mir-279/996* genomic transgene, or *mir-279*-only or *mir-996*-only transgenes, we observed comparable restoration of *nerfin-1* and *escargot* transcript levels by the various miRNAs (Fig 4H and 4I). The rescued levels were consistently slightly greater than *Canton S* but were actually similar to *mir-279/996[ex15C/+]* heterozygotes in all cases. This might reflect marginally incomplete rescue by the transgenes, or alternatively, some genetic background variation between this control and the miRNA deletion genotypes. In either case, we conclude that this dramatic, fully-penetrant, neural cell specification phenotype requires the joint activity of the miR-279 and miR-996 miRNAs, and that either miRNA suffices to direct normal development of these neurons via joint repression of shared critical target genes.

### Both miR-279 and miR-996 mediate normal adult rhythmic behavior

**Fig 5:**
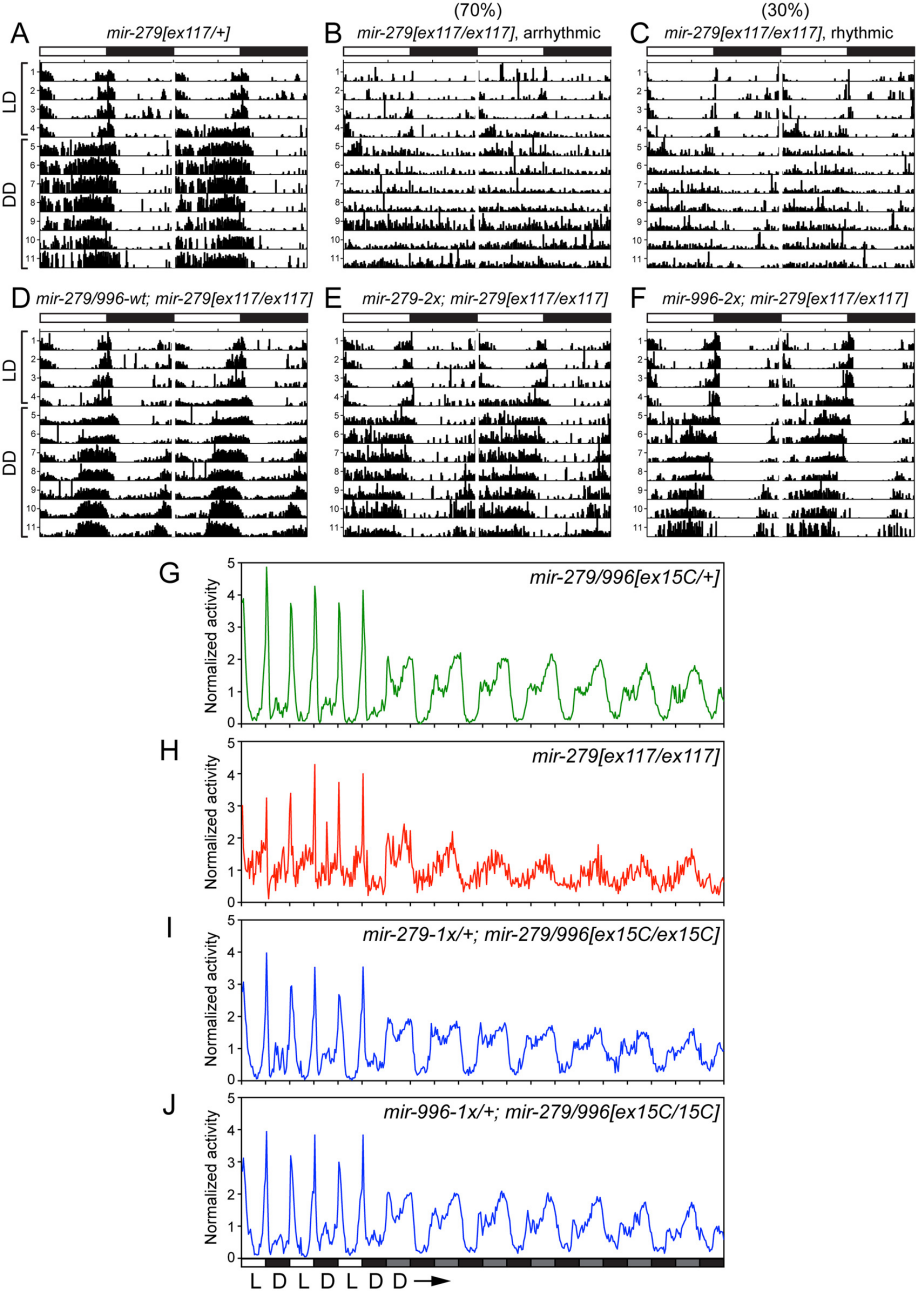
Both miR-279 and miR-996 contribute to maintenance of circadian rhythm. (A-F) Typical activity profiles of individual flies of various *mir-279/996* genotypes. Animals were entrained in 12hr-light/12hr-dark (LD) cycles for four days, and then kept in constant darkness (DD) for seven more days. In LD cycles, white bars represent the light phase (day) and black bars represent the dark phase (night). During DD cycles, grey and black bars represent the subjective day and night time, respectively. (A) *mir-279[ex117]* heterozygotes behave normally in that they maintain circadian activity in the dark, although the strong morning and evening activity peaks and mid-day siesta are not as well maintained. (B-C) In *mir-279[ex117]* homozygotes, the majority of animals gradually lost behavioral rhythmicity after transferring to constant darkness (B), but about 1/3 of animals could maintain circadian activity in constant darkness (C). Note that all *mir-279[ex117/ex117]* animals exhibited generally less activity than heterozygotes. The activity and circadian defects in *mir-279[ex117/ex117]* animals were rescued by single copies of the wild-type 16.6kb *mir-279/996* transgene (D) or the *2x-mir-279*-only (E) or *2x-mir-996*-only (F) transgenes. (G-J) Averaged activity profiles of various *mir-279/996* genotypes. (G) *mir-279/996[ex15C]* heterozygotes exhibit robust behavioral rhythmicity after transferring to constant darkness, but *mir-279[ex117]* homozygotes do not. In the *mir-279/996[ex15C]* homozygous background (which is normally mostly lethal by ~4 days), expression of only a single *1x-mir-279* (I) or single *1x-mir-996* (J) transgene can restore normal rhythmic behavior in constant darkness. n = ~32 for each genotype; the number of flies assayed for each genotype are indicated in Table 1.

We next tested the potential involvement of miR-996 in maintenance of circadian behavior. We initially studied the hypomorphic *mir-279[ex117]* homozygous condition, and confirmed previous observations [24] that a majority of *mir-279[ex117]* mutant flies displayed arrhythmic locomotor activity in constant darkness (Fig 5A and 5B). Unexpectedly, however, we further observed that 30% of mutant individuals were weakly rhythmic (Fig 5C). The quantification of percentage of rhythmic animals, their circadian period, and their power of rhythmicity are shown in Table 1. Restoration of either miR-279 or miR-996 on the *[ex117]* background, in either two doses or in a single dose, fully recovered behavioral rhythmicity (Fig 5D–5F and Table 1). The normalized activity profiles of all the different transgene combinations in the *[ex117]* background are provided in S3 Fig.

**Table 1:** Both miR-279 and miR-996 contribute to circadian rhythm.

Genotype	% of rhythmicity (n/n)	Period±SEM (hr)	Power of rhythmicity
*mir-279[ex117/+]*	100% (32/32)	23.84±0.07	105.5±5.8
*mir-279[ex36/+]*	96.9% (31/32)	23.71±0.06	109.8±5.6
*mir-279/996[ex15C/+]*	100% (32/32)	23.67±0.05	107.0±5.3
*mir-996[ex310]/+ #1*	100% (14/14)	23.61±0.08	79.3±6.5
*mir-996[ex310]/+ #2*	96.9% (31/32)	23.60±0.04	66.8±4.9
*mir-996[ex310] #1*	100% (29/29)	23.90±0.08	85.0±5.2
*mir-996[ex310] #2*	96.9% (31/32)	23.84±0.07	62.6±5.6
*mir-279[ex117/ex117]*	30.2% (16/53)	23.59±0.07	8.5±2.0
*mir-279/996-wt/+; mir-279/996[ex117/ex117]*	100% (32/32)	23.64±0.05	115.7±6.4
*mir-279-2x/+; mir-279/996[ex117/ex117]*	100% (32/32)	23.63±0.05	75.5±5.9
*mir-996-2x/+; mir-279/996[ex117/ex117]*	100% (32/32)	23.55±0.04	71.8±5.0
*mir-996-1x/+; mir-279/996[ex117/ex117]*	96.8% (30/31)	23.80±0.07	75.3±5.3
*mir-279-1x; mir-279/996[ex117/ex117]*	100% (32/32)	23.52±0.05	96.6±5.2
*mir-279/996-wt/+; mir-279/996[ex36/ex15C]*	96.9% (31/32)	23.39±0.05	89.0±6.8
*mir-279-2x/+; mir-279/996[ex36/ex15C]*	96.9% (31/32)	23.55±0.04	68.7±5.7
*mir-996-2x/+; mir-279/996[ex36/ex15C]*	100% (32/32)	23.44±0.04	96.0±4.8
*mir-996-1x/+; mir-279/996[ex36/ex15C]*	100% (32/32)	23.58±0.05	104.5±6.2
*mir-279-1x/+; mir-279/996[ex36/ex15C]*	100% (32/32)	23.41±0.04	78.4±6.7
*mir-279/996-wt/+; mir-279/996[ex15C/ex15C]*	100% (32/32)	23.36±0.04	80.1±6.3
*mir-279-2x/+; mir-279/996[ex15C/ex15C]*	93.8% (30/32)	23.55±0.04	66.8±4.9
*mir-996-2x/+; mir-279/996[ex15C/ex15C]*	100% (32/32)	23.44±0.04	78.6±5.1
*mir-996-1x/+; mir-279/996[ex15C/ex15C]*	93.8% (30/32)	23.60±0.06	64.2±5.9
*mir-279-1x/+; mir-279/996[ex15C/ex15C]*	84.4% (27/32)	23.61±0.06	51.7±4.1

Power of rhythmicity was determined by subtracting the significance line from the chi-squared power. Flies were defined as rhythmic for those with power of rhythmicity > = 10. The average period only considered rhythmic flies. For average power of rhythmicity, all living flies were included with arrhythmic flies having a value of 0.

We performed more stringent tests by asking if either miRNA could rescue the coordinate absence of both miR-279 and miR-996. Circadian rhythm assays require flies to be mobile for at least a week. Since homozygous *mir-279[ex36]* and *mir-279/996[ex15C]* adult flies are poorly able to stand or walk, and die within a few days (Fig 1C), these genotypes cannot be assayed for circadian behavior. Heterozygotes of the double deletion *[ex15C]* exhibit normal rhythmic behavior (Fig 5G); for comparison, the normalized activity pattern of surviving *[ex117]* homozygotes is shown in Fig 5H. When we assayed our panel of recombineered transgenes in *mir-279/996[ex36/ex15C]* transheterozygotes, which should be close to a null condition for the locus, we observed normal rhythmic behavior in all cases (Table 1). Finally, we performed the strictest test by assaying rescues of *[ex15C]* homozygotes. Strikingly, all transgene isoforms, including single *mir-279* and *mir-996* versions, fully restored the normal circadian clock in the mutant flies (Fig 5I and 5J and Table 1). Altogether, these data indicate that intact *mir-996* expression fully complements loss of *mir-279* in both nervous system development and adult neurophysiology.

### Similar and distinct capacities of miR-279/996 in gain-of-function analyses

**Fig 6:**
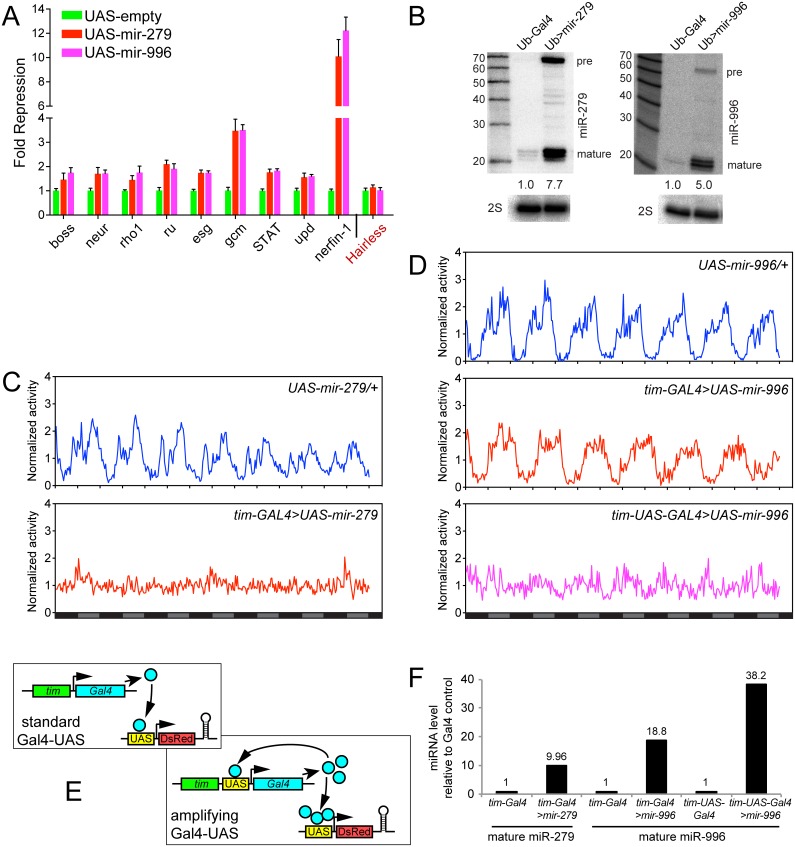
Comparison of gain-of-function activities of miR-279 and miR-996. (A) Luciferase sensor assays in S2 cells indicated that 3' UTRs of multiple miR-279 targets are all additionally responsive to miR-996. The control *Hairless* 3' UTR has no miR-279/996 seed match and was not repressed by these miRNAs. Error bars represent standard deviation from quadruplicate assays. (B) Northern confirmation of ectopic miR-279 and miR-996 in S2 cell experiments. pre = pre-miRNA hairpin, mature = mature miRNA product. Overexpressed miRNAs were calculated relative to endogenous mature miRNAs, normalized to 2S loading control. (C-D) Averaged activity profiles for control and miRNA overexpressing flies for 7 days in constant darkness since the second day after transferring from LD to DD. Some of these experiments utilized the amplifier driver *tim-UAS-Gal4*, as schematized in (E). (C) Overexpression of miR-279 by *tim-Gal4* induced strong arrhythmia. (D) Ectopic expression of miR-996 by *tim-Gal4* had no significant affect on circadian behavior, but further induction by *tim-UAS-Gal4* led to a complete arrhythmia. n = ~32 for each genotype; the number of flies assayed for each genotype is indicated in Table 2. (F) Validation that higher levels of mature miR-996 are induced by *tim-UAS-Gal4*, compared to *tim-Gal4*. Overexpressed miRNAs were quantified as in (B) using Northern blotting, and normalized to 2S loading control. These tests also confirm that *tim-Gal4*>*UAS-mir-996* flies effectively misexpressed miR-996, even though they lacked circadian defects.

The evidence gathered indicates that miR-279 and miR-996 play surprisingly similar roles in diverse developmental and behavioral settings. Nevertheless, their distinct conserved 3' sequences (Fig 1B) suggests that these miRNAs have diversified in some way during evolution. As we showed in the head, endogenous miR-279 and miR-996 exhibit comparable activity to restrict the accumulation of *nerfin-1* and *escargot* transcripts (Fig 4H). To probe this further, we compared the gain-of-function activities of these miRNAs using luciferase sensor assays in S2 cells. We tested 3' UTR sensors for nine genes bearing conserved miR-279 family target sites, including all of those identified in previous in vivo studies. Transfection of *ub-Gal4* and *UAS-dsRed-miRNA* expression constructs led to significant repression of all sensors (Fig 6A). As a negative control, the *Hairless* 3' UTR does not contain any miR-279/996 binding site and the sensor was not repressed upon miRNA transfection (Fig 6A). We verified comparable ectopic expression of both miR-279 and miR-996 in these transfection experiments (Fig 6B). Therefore, the capacities of miR-279 and miR-996 in S2 cells are similar.

To explore their ectopic activities *in vivo*, we overexpressed the miRNAs in the circadian neurons with *tim-Gal4*. In accordance with previous studies [24], overexpression of miR-279 in circadian tissues strongly disrupted the adult behavioral rhythm. In contrast to the unactivated transgene background, *tim>mir-279* animals quickly became arrhythmic following their transfer to constant darkness (Fig 6C). However, ectopic miR-996 only weakly affected circadian rhythm, with 13% arrhythmic flies observed with only one of the two insertions (Fig 6D). Quantitative data for these genotypes is shown in Table 2. This was not due to inability to produce miR-996, since the degree of accumulation of ectopic miR-996 induced by *tim-Gal4* was greater than for miR-279 (Fig 6F). Such phenotypic differences suggested that miR-279 has stronger capacity to influence circadian cell activity, even though endogenous *mir-996* is completely able to compensate for the absence of *mir-279*.

**Table 2:** Circadian rhythm defects upon overexpression of miR-279 family miRNAs.

Genotype	% of rhythmicity (n/n)	Period±SEM (hr)	Power of rhythmicity
*tim-Gal4/+*	100% (10/10)	24.30±0.15	82.3±11.6
*tim-UAS-Gal4/+*	93.3% (14/15)	23.82±0.10	96.0±10.6
*UAS-mir-279/+*	93.8% (15/16)	23.20±0.07	75.4±10.5
*UAS-mir-996(#7721)/+*	93.8% (15/16)	23.50±0.00	114.8±15.3
*UAS-mir-996(#7722)/+*	92.9% (13/14)	23.50±0.00	110.4±11.5
*tim-Gal4/UAS-mir-279*	0% (0/29)	N/A	0
*tim-Gal4/UAS-mir-996(#7721)*	86.7% (13/15)	24.85±0.07	63.4±12.1
*tim-Gal4/UAS-mir-996(#7722)*	100% (14/14)	24.61±0.09	111.7±10.4
*tim-UAS-Gal4/UAS-mir-996(#7721)*	18.8% (3/16)	23.33±0.61	6.1±3.1
*tim-UAS-Gal4/UAS-mir-996(#7722)*	56.3% (9/16)	23.83±0.22	30.8±9.6

Power of rhythmicity was determined by subtracting the significance line from the chi-squared power. Flies were defined as rhythmic for those with power of rhythmicity > = 10. The average period only considered rhythmic flies. For average power of rhythmicity, all living flies were included with arrhythmic flies having a value of 0.

Nevertheless, given the substantial overlapping capacities of these miRNAs for target regulation (Fig 6A), we asked whether increased levels of *mir-996* could influence circadian rhythm. For these tests, we utilized *tim-UAS-Gal4*, which auto-potentiates Gal4 expression in *tim*-expressing neurons (Fig 6E). We used Northern blotting to verify that more mature miR-996 was generated in the latter condition (Fig 6F). Interestingly, in this sensitized overexpression background, miR-996 induced substantial behavioral arrhythmia (81% and 44% of the independent *UAS-mir-996* insertions exhibited arrhythmia, Fig 6D and Table 2). In summary, these gain-of-function experiments reveal intrinsic differences between miR-279 and miR-996, which otherwise exhibit surprising genetic redundancy under carefully controlled endogenous conditions. This is perhaps counter to normal expectation, in which overexpressed seed family miRNAs more typically exhibit similar properties even on the transcriptome level [35,36], but may instead display functional distinctions under physiological settings.

## Discussion

### miRNAs with diverse and profound endogenous phenotypic requirements

Despite extensive experimental and computational evidence for the pervasive nature of animal miRNA target networks, genetic studies have not generally supported the notion that animals rely upon miRNAs to the same degree, as say, transcription factors and signaling pathways [37]. This was strikingly evident with the systematic knockout of *C*. *elegans* miRNAs, which revealed barely any developmental or behavioral phenotypes [12]. Similarly, a genomewide collection of *D*. *melanogaster* miRNA knockouts reveals a variety of phenotypes, but these are generally quantitative in nature and include few documented developmental defects [38]. While this might partly be due to functional overlap amongst members of similar miRNA families, compound knockouts of *C*. *elegans* miRNA families revealed overt consequences only for a minority of families [39,40]. Only upon further sensitization, by reducing the activity of other gene broad regulators, did additional miRNA knockouts exhibit phenotypes [41]. Together with studies of dozens of miRNAs that mostly exhibit phenotypes under sensitized conditions [42–44], an emerging concept is that miRNAs mostly act as robustness factors [5,16].

Nevertheless, select miRNAs have proven to be essential for certain development or physiological processes. Our current studies affirm and extend the broad impact of the *mir-279*/*mir-996* locus, which generates phenotypically critical miRNAs of profound impact. Together, these miRNAs are fully essential for organismal viability, for normal cell specification of olfactory neuron subtypes, and for rhythmic behavior via circadian pacemaker cells. An unexpected conclusion of this work was to uncover that these two miRNAs, previously inferred to derive from separate transcription units [26], actually provide highly overlapping in vivo activities. Although the stringent evolutionary conservation of these miRNAs is *de facto* evidence that they are not truly "redundant", we demonstrate using precise genetic engineering that single genomic copies of either *mir-279* or *mir-996* can fully compensate for the deletion of all four miRNA alleles in diverse developmental and physiological settings.

A notable feature of the function of this miRNA family operon is that they mediate their effects through multiple key, setting-specific, targets. For example, the transcription factors encoded by *nerfin-1* and *escargot* are the critical miR-279/996 targets whose de-repression induces ectopic CO_2_-sensing neurons, and whose heterozygosity confers substantial rescue of ectopic CO_2_-sensing neurons in flies that lack these miRNAs [18,23]. On the other hand, miR-279/996 have substantial effects on different aspects of the JAK-STAT signaling pathway in circadian pacemaker cells and ovarian border cells, by targeting the ligand unpaired [24] and the transcription factor STAT [25], respectively.

While manipulation of these various targets can substantially rescue setting-specific phenotypes caused by loss of miR-279 and miR-996, none of them rescue the extremely abbreviated lifespan of the double mutant. It remains to be seen whether this marked phenotype is due to combined de-repression of multiple characterized targets, or to a different pathway or target network. Our newly characterized *mir-279/996* alleles and rescue backgrounds comprise valuable reagents for future study of these miRNAs.

### Dominance of seed sequences for *in vivo* miRNA functions

Early genetic studies revealed the principle of miRNA seed targeting [10,11], many years before the complementary miRNAs were identified [9,45]. Since then, a wealth of experimental studies have shown that ~7 nt seed matches are sufficient to confer substantial regulation by miRNAs, not only in culture cells [46] but also in the animal [47,48]. Genomewide studies show that seed-matching is the dominant mode of conserved target recognition [49–51]. In addition, overexpression tests clearly demonstrate that the dominant transcriptome signature induced by ectopic miRNAs is seed-based, and maintained even upon substitution of the remainder of the miRNA sequence [35,36].

Nevertheless, considerable debate continues about the contribution of "non-seed" target sites to miRNA networks. Notably, some of the earliest miRNA targets found, which definitively mediate regulatory interactions critical for development, lack continuous seed matching [7,8]. In the classic example of let-7:*lin-41* pairing, the atypical architecture of a bulged seed supplemented by extended 3' pairing is proposed to permit specific recognition that cannot be achieved by other let-7 family members in C. elegans [40,52]. A number of subsequent directed studies define functional and/or conserved miRNA:target pairing configurations, characterized by 3' compensatory pairing, distributive complementarity, or centered pairing [47,53,54]. The existence of such sites has been attractive from the point of view that they might help explain strong evolutionary constraints on entire miRNA sequences, which are not satisfactorily explained by seed regions alone. However, it remains to be seen whether mutations of non-seed miRNA sequences are of consequence to *in vivo* miRNA-mediated phenotypes.

On the other hand, available genetic studies suggest limits on the contribution of non-seed regions to *in vivo* miRNA phenotypes. For example, all known miRNA point mutant alleles, other than ones that broadly affect biogenesis, invariably prove to alter seed regions [6,8,21,22,55,56]. In addition, Horvitz and colleagues showed that embryo/larval lethality caused by compound mutations of the 8-member *mir-35* family or the 6-member *mir-51* family could both be rescued by individual family members [39]. Our studies of miR-279 and miR-996 provide a new testbed for this, since this locus is responsible for several of the most overt developmental and behavioral phenotypes ascribed to animal miRNAs. While we provide functional evidence that these miRNAs are not identical in regulatory capacity and/or processing, it is still striking from the biological viewpoint that multiple essential *in vivo* phenotypes can be fully satisfied by either of these non-seed divergent miRNAs.

It should be informative to conduct similar studies for some of the other miRNAs that exhibit overt phenotypic requirements, potentially by systematic mutation of 3' regions, to assess their relative contribution to organismal phenotypes. With the advent of CRISPR/Cas9 genome engineering, it is now feasible to address these questions with respect to miRNA genes and miRNA sites in the genomes of intact organisms [57,58].

## Materials and Methods

### Generation of new *mir-279/996* deletion alleles

The *mir-996* single deletion and *mir-279/996* double deletion alleles were generated by imprecise excision of *P{EPgy2}CR31044[EY03350]*, which is inserted 370bp downstream of the *mir-996* hairpin. We crossed these to the *TMS*, *Δ2-3/TM6B* jumpstarter and induced transposition in *P{EPgy2}CR31044[EY03350]*/*TMS*, *Δ2–3* animals. Following the segregation of *TMS*, *Δ2–3*, we screened ~500 candidate excision chromosomes for deletions in the *mir-279*/*mir-996* region using the the following PCR amplicons: mir279F excision CAAGAAACCACCCCGAGAAGAAGAAG mir279R excision AGCAGGTGTTACAGTTACACTCAAACG.

The *mir-996[ex310]* deletion contains a 568 deletion with 9bp of P-element sequence left, while the *mir-996[ex187]* deletion contains a 584 bp deletion with 154bp of P-element sequence left. The *mir-279/996[ex15C]* allele bears a 2825bp deletion that removes both miRNA hairpins and retains 84bp of P-element sequence.

### Generation of *mir-279/996* genomic rescue transgenes

The 3.0kb genomic sequence containing only *mir-279* was cloned from the genome and inserted into the pBDP vector [59]. For the large rescue transgenes, we retrieved 16.6kb extending into both upstream and downstream protein-coding genes of the *mir-279/996* locus from the BAC CH322-35G11 (BACPAC Resources) and cloned it into the attB-P[acman]-AmpR vector by recombineering as described [60]. The *mir-279* or *mir-996* hairpins were targeted with an *rpsL-neo* cassette (Gene Bridges), which was flanked by the ~50bp left and right homology arms for the miRNA and carried two *BbvCI* restriction sites between the *rpsL-neo* cassette and the homology arms. We then deleted the *rpsL-neo* cassette from the targeted construct by BbvCI digestion and the remaining vector was re-ligated to generate the *mir-279-1x* or *mir-996-1x* construct.

To generate the *mir-279-2x* construct, genomic fragments 13.5kb upstream and 3.0kb downstream of the *mir-996* hairpin were retrieved from the CH322-35G11 BAC and cloned between the *AscI* and *NotI* sites of the attB-P[acman]-AmpR vector. The 5’ end of the upstream and 3' end of the downstream fragment were identical to the ends of the 16.6kb wild type genomic fragment. The *mir-279* hairpin was PCR cloned and inserted to the 5' end of the *mir-996* downstream fragment, then the resultant 3.1kb piece was digested out and ligated with the 13.5kb *mir-996* upstream sequence to generate the *mir-279-2x* construct. Similar procedures were followed to generate the *mir-996-2x* construct. Such *mir-279-2x* and *mir-996-2x* constructs carried a *NotI* site at the 5' side and an *AscI* site at the 3' side of the ectopic hairpin. Sequences of the primers used are listed in S1 Table. Transgenes were generated using the phiC31 system (BestGene Inc).

### Other *Drosophila* mutants and transgenes

The *mir-279[ex117]* (also known as *Δ1*.*2*) and *mir-279[ex36]* (also known as *Δ1*.*9*) alleles were generated in the lab previously [18], but outcrossed stocks were obtained from Amita Sehgal [24] and used in this study. Other previously-described stocks utilized in this study include *UAS-luc-mir-279* and *UAS-DsRed-mir-996* [61], *tim-Gal4* and *tim-UAS-Gal4* [62], and the MARCM tester stock *eyflp; Gr21-Gal4*, *UAS-sytGFP; FRT82*, *tubGal80* [18,63].

### Small RNA northern blot

Total RNAs were extracted using Trizol LS (Life Technologies) following the manufacturer’s protocol. RNA samples were separated on 12% polyacrylamide denaturing gels (National Diagnostics), transferred to the GeneScreen Plus (Perkin Elmer) membrane, crosslinked with UV light and hybridized with γ-^32^P-labeled LNA (Exiqon) antisense probes for miR-279 and miR-996 at 45°C overnight. Signals were exposed to an Imaging Plate (Fujifilm) for 2~3 days for appropriate signal intensity. 2S RNA was hybridized with DNA probe and exposed for 30 min as the loading control. Signal quantifications were performed in the Image Gauge software and levels of miR-279 and miR-996 in different genotypes were normalized to 2S rRNA.

### Lifespan assay

Late stage pupae of *mir-279[ex117]*, *mir-279[ex36]* and *mir-279/996[ex15C]* mutants were transferred from culture bottles to clean petri dishes humidified with wet Kimwipe papers. This was necessary because of the overall poor vigor of the mutant stocks. For flies carrying rescue transgenes, no extra care was needed as all wildtype and modified constructs fully restored normal robustness of the mutant backgrounds. Adult flies of all genotypes were collected within 24 hours of eclosion into normal food vials and maintained in room temperature (22°C) in low density (10 flies per vial). Flies were transferred to fresh vials every day for mutants and every 3 to 5 days for rescued genotypes and scored for survivors across a time frame of 20 days. Again, the additional care was necessary to extend the lifespan of the mutants, which would normally succumb much more prematurely due to becoming trapped in the food.

### Immunohistochemistry

Adult heads were fixed on ice for 2~3 hours in the fixative solution containing 4% paraformaldehyde (PFA) and 0.2% Triton-X-100 in PBS (0.2% PBST). Heads were then rinsed with 0.2% PBST for 3 times and brains were dissected in the blocking solution (5% normal goat serum in 0.2% PBST). Both primary and secondary antibodies were incubated overnight at room temperature. Brains were washed 3 times with 0.2% PBST before and after the secondary antibody incubation and mounted in the Vectashield mounting medium with DAPI (H-1200, Vector Labs). Antibodies were used as follows: mouse-anti-nc82 (1:20, Developmental Studies Hybridoma Bank), rabbit-anti-GFP (1:1000, Invitrogen), and Alexa Fluor-488, -568 secondary antibodies (1:500, Molecular Probes).

### Circadian behavior assay

Flies were entrained in 12-hour light/12-hour dark (LD) cycles at 25°C for 5 days before transferred into constant darkness (DD) during the dark phase of the LD cycle. Locomotor activities of individual flies were recorded with the Drosophila Activity Monitor System (TriKinetics) every 1 min, data were then binned at 30 min with the DAMFileScan and the circadian period was calculated using ClockLab (Actimetrics) from data collected for 7 days in DD conditions. The power of rhythmicity was calculated as the chi-squared power above the significance line [64].

### Luciferase assay

The 3' UTRs of *Hairless* and predicted miR-279 target genes were cloned between the *XhoI* and *NotI* sites of a modified psiCHECK-2 vector [65]. Sensor plasmid and *Ub-Gal4* were cotransfected with *UAS-dsRed-miRNA* [61] or empty *pUAST* vector into S2-R+ cells with the Effectene (Qiagene) reagents. Luciferase levels were measured using the Dual-Glo Luciferase Assay System (Promega). Primer sequences for 3' UTR cloning are listed in S1 Table.

### Gene expression analysis

We prepared cDNA from Trizol-extracted RNA that was treated with DNase and reverse transcribed using QuantiTect Reverse Transcription Kit (Qiagen). qPCR reactions were performed using SYBR select master mix (Life Technologies). Data were normalized to *Rpl32* amplification. Primer sequences for qPCR amplicons are listed in S1 Table.

## Supporting Information

S1 Fig Coexpression of miR-279 and miR-996. Shown are analyses from deeply-sequenced libraries from various Drosophila stages and tissues (Ruby et al, Genome Research 2007). The accumulation of miR-286 is largely restricted to the early to mid embryo stages, and is nearly absent thereafter. miR-279 and miR-996 co-accumulate at various embryonic and post-embryonic settings.
(PDF)

S2 Fig Severe loss of mature miR-996 expression in *mir-279* deletion alleles. Shown are Northern blots of miR-279 and miR-996 in various *mir-279* and *mir-996* homozygous or trans-heterozygous allele combinations. These experiments utilized male body and male head RNA samples, and show similar results as to female samples shown in main Fig 2. Levels of mature miR-996 are strongly diminished in the *mir-279* alleles *[ex117]* and *[ex36]* that retain the *mir-996* genomic DNA. *mir-996[ex310]* is a deletion of the *mir-996* region that does not affect *mir-279*, and *mir-279/996[ex15C]* is a deletion of both miRNAs.
(PDF)

S3 Fig Normalized activity profiles of *mir-279/996* transgene rescues of *mir-279[ex117]* mutants. Shown are normalized activity profiles of various *mir-279[ex117]* genotypes following entrainment in 12 hour light/12 hour dark (LD) cycles, then assayed for circadian behavior in constant darkness (DD). A minimum of 20 individuals were analyzed for each genotype. The *[ex117]* allele, which is null for miR-279 and strongly hypomorphic of miR-996, exhibits normal circadian behavior as a heterozygote (A) but not as a homozygote (B). The rhythmic behavior of *[ex117]* homozygotes was fully rescued by a single insertion of the wildtype 16.6 kb genomic transgene covering the *mir-279/996* locus (C). Circadian activities were also recovered by each member of a mutant transgene panel (as detailed in the inset box) bearing reciprocal substitutions of *mir-279* or *mir-996* into the other hairpin locus (*mir-279-2x*, D and *mir-996-2x*, E), as well as by knockout transgenes for either miRNA (*mir-279-1x*, F and *mir-996-1x*, G).
(PDF)

S1 Table Primer sequences used to clone *mir-279/996* rescue transgenes and *mir-279/996* luciferase sensor constructs, and quantify target genes. (PDF)
