# Supplementary figures and images for: Multiple In Vivo Biological Processes Are Mediated by Functionally Redundant Activities of Drosophila mir-279 and mir-996
Source: PLoS Genet. 2015 Jun 4;11(6):e1005245. doi: 10.1371/journal.pgen.1005245 (PMC4456407; doi:10.1371/journal.pgen.1005245)

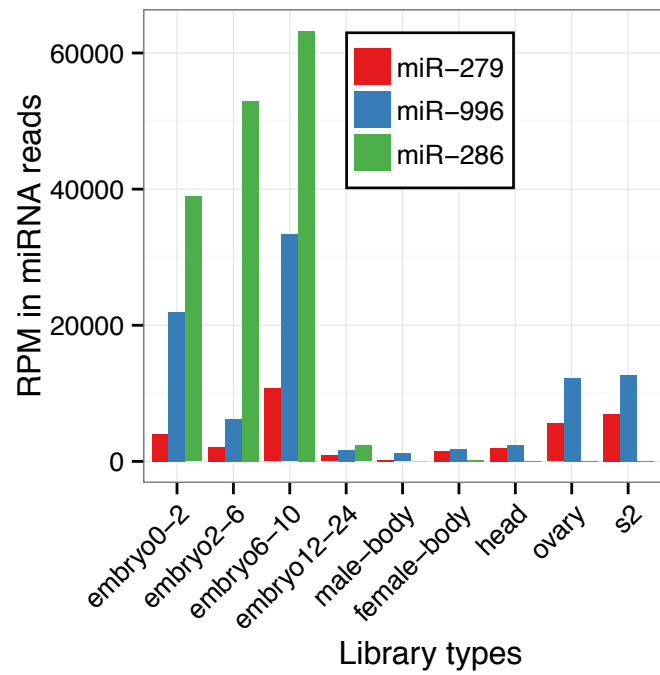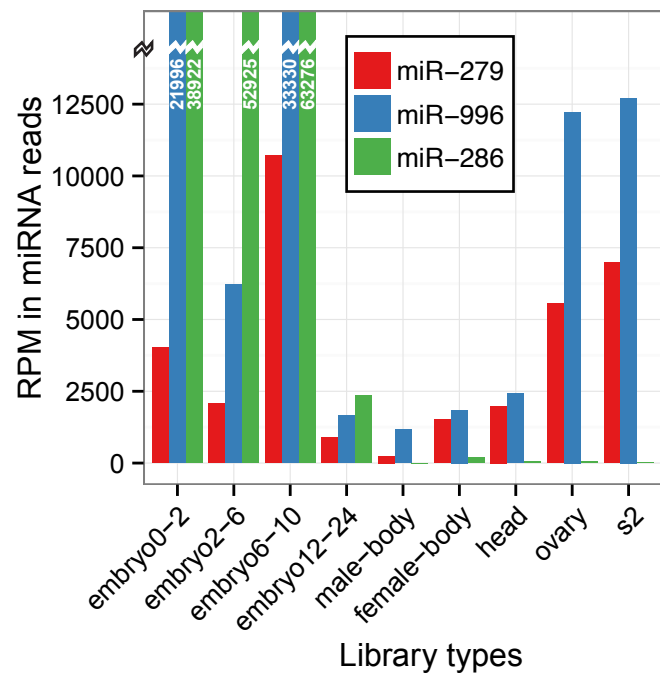

Sun et al  
Supplementary Figure 1

Supplement: S1 Fig — Shown are analyses from deeply-sequenced libraries from various Drosophila stages and tissues (Ruby et al, Genome Research 2007). The accumulation of miR-286 is largely restricted to the early to mid embryo stages, and is nearly absent thereafter. miR-279 and miR-996 co-accumulate at various embryonic and post-embryonic settings. (PDF) [file pgen.1005245.s001.pdf]

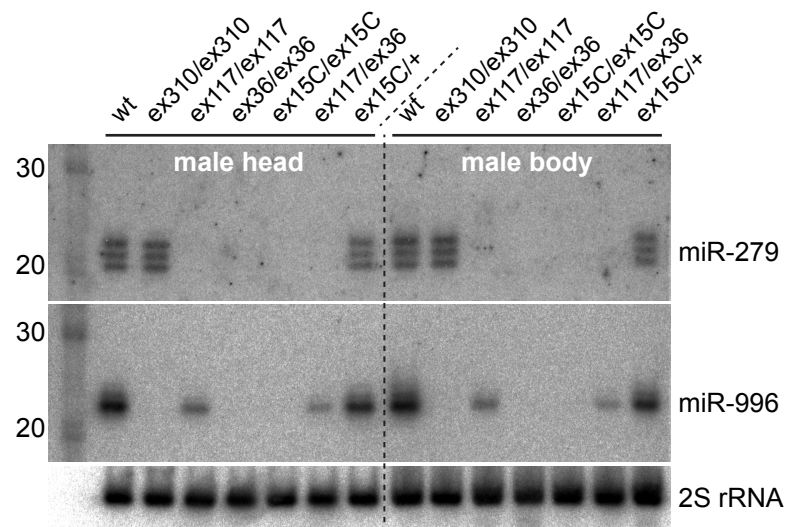

Sun et al  
 Supplementary Figure 2

Supplement: S2 Fig — Shown are Northern blots of miR-279 and miR-996 in various mir-279 and mir-996 homozygous or trans-heterozygous allele combinations. These experiments utilized male body and male head RNA samples, and show similar results as to female samples shown in main Fig 2. Levels of mature miR-996 are strongly diminished in the mir-279 alleles [ex117] and [ex36] that retain the mir-996 genomic DNA. mir-996[ex310] is a deletion of the mir-996 region that does not affect mir-279, and mir-279/996[ex15C] is a deletion of both miRNAs. (PDF) [file pgen.1005245.s002.pdf]

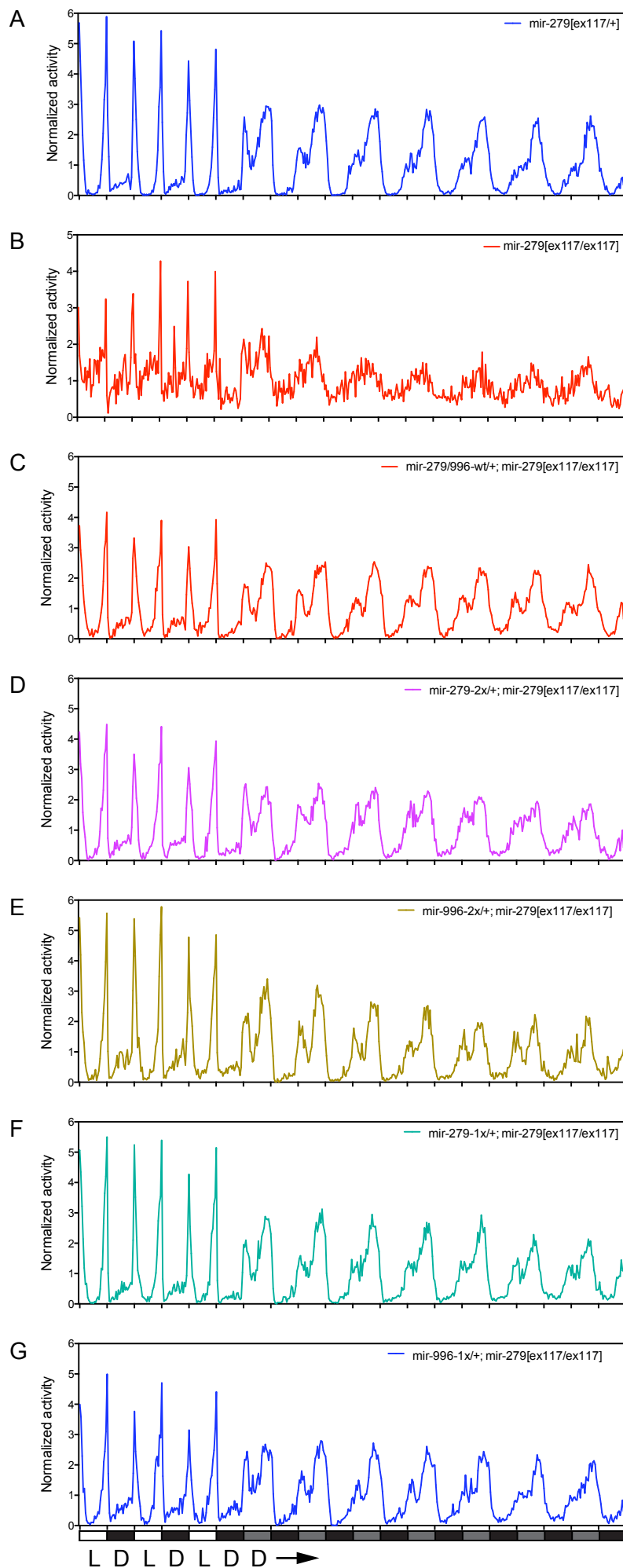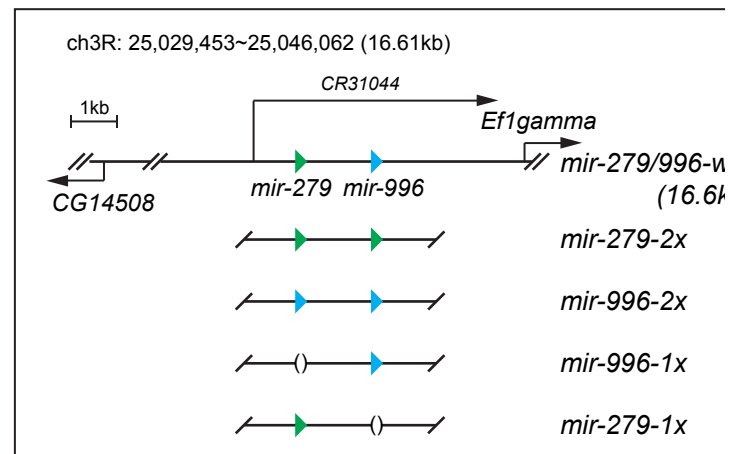

Supplement: S3 Fig — Shown are normalized activity profiles of various mir-279[ex117] genotypes following entrainment in 12 hour light/12 hour dark (LD) cycles, then assayed for circadian behavior in constant darkness (DD). A minimum of 20 individuals were analyzed for each genotype. The [ex117] allele, which is null for miR-279 and strongly hypomorphic of miR-996, exhibits normal circadian behavior as a heterozygote (A) but not as a homozygote (B). The rhythmic behavior of [ex117] homozygotes was fully rescued by a single insertion of the wildtype 16.6 kb genomic transgene covering the mir-279/996 locus (C). Circadian activities were also recovered by each member of a mutant transgene panel (as detailed in the inset box) bearing reciprocal substitutions of mir-279 or mir-996 into the other hairpin locus (mir-279-2x, D and mir-996-2x, E), as well as by knockout transgenes for either miRNA (mir-279-1x, F and mir-996-1x, G). (PDF) [file pgen.1005245.s003.pdf]
