# Supplementary material for: Multiple In Vivo Biological Processes Are Mediated by Functionally Redundant Activities of Drosophila mir-279 and mir-996
Source: PLoS Genet. 2015 Jun 4;11(6):e1005245. doi: 10.1371/journal.pgen.1005245 (PMC4456407; doi:10.1371/journal.pgen.1005245)
Supplement: S1 Table — (PDF) [file pgen.1005245.s004.pdf]

Supplementary Table 1. Primers for mir-279/996 rescue constructs and sensor constructs		
generation of 3.4kb rescue construct		NOTE
NotI-mir279-3.4k-Fwd	ATAAGAATGCGGCCGCTGAAAATACGCGTATGGAAATGCC	
BglII-mir279-3.4k-Rev	GAAGATCTCAGAATCGGAATCGGAATCAG	
generation of 16.6kb wild type construct		
Asc-16.6kb-5'LA-Fwd	TTGGCGCGCCGCCATTTTCGGTCGGATTCAG	~600bp left arm
BamH-16.6kb-5'LA-Rev	AGCAGCGAACTATGTTAAGATGGAAGGGATCCCATCCTGCCTTCTCTGTAGTTCCAG	
BamH-16.6kb-3'RA-Fwd	CTGGAACTACAGAGAAGGCAGGATGGGATCCCTTCCATCTTAACATAGTTCGCTGCT	~600bp right arm
Not-16.6kb-3'RA-Rev	ATAGTTTAGCGGCCGCGGAAAATCCTCACTGCCCTTGTG	
generation of mir-279-1x and mir-996-1x constructs		
Bbv-mir279-LA-rpsLneo-Fwd	GAATTGGGACTGGAGCTGGAATTCCTCAGCGGCCTGGTGATGATGGCGGGATCG	~50bp left arm for mir-279
mir279-LA-Fwd2	AATGGAGAACGCAAAAATCCCATTATAATGGAATTGGGACTGGAGCTGGAATT	
Bbv-mir279-RA-rpsLneo-Rev	GTCAAGAAAACACGTGCTTGATGATTGCTGAGGTCAGAAGAACTCGTCAAGAAGGCG	~50bp right arm for mir-279
mir279-RA-Rev2	CTTTTCGAATGAAGTACACGCGAAGTTTGTCAAGAAAACACGTGCTTGATGATT	
Bbv-mir996-LA-rpsLneo-Fwd	GGATGTGGGGAAGGATGCTCTTTCCTCAGCGGCCTGGTGATGATGGCGGGATCG	~50bp left arm for mir-996
mir996-LA-Fwd2	CATCCGGGCGAGAAAGGAGCCCAATAGTGGGGATGTGGGGAAGGATGCTCTTT	
Bbv-mir996-RA-rpsLneo-Rev	AAAGCTCCGCTCTCTTCGTTGTGGCTGAGGTCAGAAGAACTCGTCAAGAAGGCG	~50bp right arm for mir-996
mir996-RA-Rev2	AAAACGACTCCCTCCGATCCCCGCGAGATGGAAAGCTCCGCTCTCTTCGTTGTG	
generation of mir-279-2x construct		
Asc-13.5kb-5'LA-Fwd	TTGGCGCGCCGCCATTTTCGGTCGGATTCAG	~600bp left arm for mir-996 13.5kb upstream fragment
BamH-13.5kb-5'LA-Rev	GGGCACAAAAACAGAGAGCAGAGGGATCCCATCCTGCCTTCTCTGTAGTTCCAG	
BamH-13.5kb-3'RA-Fwd	CTGGAACTACAGAGAAGGCAGGATGGGATCCCTCTGCTCTCTGTTTTTGTGCCC	~600bp right arm for mir-996 13.5kb upstream fragment
Not-13.5kb-3'RA-Rev	ATAGTTTAGCGGCCGCAAAGAGCATCCTTCCCCACATCC	
Asc-3.0kb-5'LA-Fwd	TTGGCGCGCCACAACGAAGAGAGCGGAGCTTTC	~600bp left arm for mir-996 3.0kb downstream fragment
BamH-3.0kb-5'LA-Rev	AGCAGCGAACTATGTTAAGATGGAAGGGATCCACAACGACAACAACAACCCTCGC	
BamH-3.0kb-3'RA-Fwd	GCGAGGGTTGTTGTTGTCGTTGTGGATCCCTTCCATCTTAACATAGTTCGCTGCT	~600bp right arm for mir-996 3.0kb downstream fragment
Not-3.0kb-3'RA-Rev	ATAGTTTAGCGGCCGCGGAAAATCCTCACTGCCCTTGTG	
Asc-Not-mir279-Fwd	TTGGCGCGCCTAGCGGCCGCGGAATTCATACTACTGTTTTTAGTGGGTG	cloning of mir-279 hairpin
Asc-mir279-Rev	TTGGCGCGCCGAACTACCGTTATTAATGAGTGTGGATCT	
generation of mir-996-2x construct		
Asc-11.8kb-5'LA-Fwd	TTGGCGCGCCGCCATTTTCGGTCGGATTCAG	~600bp left arm for mir-279 11.8kb upstream fragment
BamH-11.8kb-5'LA-Rev	GCCCTCTTGTTGTTGTGTTCTTCTTCGGATCCCATCCTGCCTTCTCTGTAGTTCCAG	
BamH-11.8kb-3'RA-Fwd	CTGGAACTACAGAGAAGGCAGGATGGGATCCGAAGAAGAACACAACAACAAGAGGGC	~600bp right arm for mir-279 11.8kb upstream fragment
Not-11.8kb-3'RA-Rev	ATAGTTTAGCGGCCGCCAATTCCAGCTCCAGTCCCAATTC	
Asc-4.7kb-5'LA-Fwd	TTGGCGCGCCGTTCAATCATCAAGCACGTGTTTTC	~600bp left arm for mir-279 4.7kb downstream fragment
BamH-4.7kb-5'LA-Rev	AGCAGCGAACTATGTTAAGATGGAAGGGATCCGGAAACAAAAGGGGACTGACTCAG	
BamH-4.7kb-3'RA-Fwd	CTGAGTCAGTCCCCTTTTGTTTCCGGATCCCTTCCATCTTAACATAGTTCGCTGCT	~600bp right arm for mir-279 4.7kb downstream fragment
Not-4.7kb-3'RA-Rev	ATAGTTTAGCGGCCGCGGAAAATCCTCACTGCCCTTGTG	
Asc-Not-mir996-Fwd	TTGGCGCGCCTAGCGGCCGCTCTGACTCTATTTTGTCGGCGAACAT	cloning of mir-996 hairpin
Asc-mir996-Rev	TTGGCGCGCCCTGACCCAACTTAATAGACGAGCATGA	
NOTE:<br>Primers "Asc-16.6kb-5'LA-Fwd", "Asc-13.5kb-5'LA-Fwd" and "Asc-11.8kb-5'LA-Fwd" are the same.<br>Primers "Not-16.6kb-3'RA-Rev", "Not-3.0kb-3'RA-Rev" and "Not-4.7kb-3'RA-Rev" are the same.<br>Therefore, all large rescue transgene have roughly the same size of 16.6kb and the same 5' and 3'ends.		
Primers for luciferase sensor constructs		
Xho-boss-Psi-Fwd	CCGCTCGAGATCCACAACGTCCTCCACCT	
Not-boss-siR1	ATAGTTTAGCGGCCGCTCTTGGTTCGTCAGTGATT	
Xho-esg-Psi-Fwd	CCGCTCGAGGAACTCAACTACGCCGGCTAT	
Not-esg-siR1	ATAGTTTAGCGGCCGCGTATTTCCTTCTTTCTTCGCTTGATG	
Xho-gcm-Psi-Fwd	CCGCTCGAGGCTAGGGGATCGGGATAAGAT	
Not-gcm-siR1	ATAGTTTAGCGGCCGCGTACTCACAATTGCCATAGTTCGG	
Xho-nerfin-Psi-Fwd	CCGCTCGAGCATGGCCCACTGAAATCGAG	
Not-nerfin-siR1	ATAGTTTAGCGGCCGCCAGTTTTGCGTTTTTATTCAAGAGCTT	
Xho-neur-Psi-Fwd	CCGCTCGAGCTACACCACGTAGAAAGGTC	
Not-neur-siR1	ATAGTTTAGCGGCCGCTCCGCTTTGCTTGTCCTTCA	
Xho-rho-Psi-Fwd	CCGCTCGAGGAGATCGAGAGACAGAGAGT	
Not-rho-siR1	ATAGTTTAGCGGCCGCTGTAGGGGATTACGATGCTC	
Xho-ru-Psi-Fwd	CCGCTCGAGGCAGCATCTGATGAATGACC	
Not-ru-siR1	ATAGTTTAGCGGCCGCTAAGCCTAAGTAGGCAACTC	
Xho-STAT-Psi-Fwd	CCGCTCGAGGTGAAACGCCAAAACACAAGAAC	
Not-STAT-siR1	ATAGTTTAGCGGCCGCGGGGTGTACTTAAGTCTTATAAAATTC	
Xho-upd-Psi-Fwd	CCGCTCGAGCGTGAAATAGCATACACAACATTCC	
Not-upd-siR1	ATAGTTTAGCGGCCGCGTTCGCTTTATTCCGCTGCTG	
Primers for qPCR assays		
Nerfin1 fw	AAGCGTAAATTGAGCCGGGA	
Nerfin1 rev	CATCGAACTTCAGCTTGCGG	
Escargot fw	ATCACCATGCGCCTATCTCG	
Escargot rev	CGGCTGGTTCTTGTCCTTCT	
Rpl32 fw	CCGCTTCAAGGGACAGTATC	
Rpl32 rev	ACGTTGTGCACCAGGAACTT	
